# Examining the response programming function of the Quiet Eye: Do tougher shots need a quieter eye?

**DOI:** 10.1007/s10339-017-0841-6

**Published:** 2017-10-23

**Authors:** Rosanna Walters-Symons, Mark Wilson, Andre Klostermann, Samuel Vine

**Affiliations:** 1 University of Exeter, Exeter, UK; 2 University of Bern, Bern, Switzerland

**Keywords:** Quiet Eye, Pre-programming, Online control, Task difficulty, Golf putting

## Abstract

Support for the proposition that the Quiet Eye (QE) duration reflects a period of response programming (including task parameterisation) has come from research showing that an increase in task difficulty is associated with increases in QE duration. Here, we build on previous research by manipulating three elements of task difficulty that correspond with different parameters of golf-putting performance; force production, impact quality and target line. Longer QE durations were found for more complex iterations of the task and furthermore, more sensitive analyses of the QE duration suggest that the early QE proportion (prior to movement initiation) is closely related to force production and impact quality. However, these increases in QE do not seem functional in terms of supporting improved performance. Further research is needed to explore QE’s relationship with performance under conditions of increased difficulty.

## Introduction

The Quiet Eye (QE; Vickers 1996)—defined as the final fixation directed to a single location or object prior to initiation of movement—has become a well-established characteristic of expertise and proficiency (for a recent meta-analysis and review see Lebeau et al. 2016). However, there is a lack of clarity in the literature regarding the potential mechanisms through which it exerts its influence. The predominant explanation is that the QE reflects a period of response programming, where task parameterisation (e.g. force and direction) occurs as a result of the consolidation of information from the QE duration itself, as well as previous fixations and performance attempts (for a recent overview see Gonzalez et al. 2015).

Several noteworthy attempts have been made to experimentally examine the response programming function of the QE by manipulating task difficulty in billiards shooting (Williams et al. 2002) and in ball (Klostermann et al. 2013) and dart (Horn et al. 2012) throwing tasks. In each case, longer QE durations were found when tasks place greater demands on response programming. However, as well as some equivocal findings—Wilson and Pearcey (2009) found that QE duration in golf putting was not associated with changes in the slope of the putting surface—previous research has been imprecise in how task difficulty has been manipulated. For instance, Williams et al. (2002) focused on manipulating the complexity of well-known billiards shots that involved the programming of different shot angles, which may not reflect QE’s response to other relevant parameters such as changes in force production.

The first aim of this experiment was therefore to examine the influence of manipulations of task difficulty that correspond with different parameters of golf-putting performance, e.g. force production, impact quality and target line (Pelz 2000) on the QE duration adopted by experienced golfers. We  predicted that increased task difficulty, requiring more detailed and specific parameterisation, should be associated with longer QE durations. Specifically, longer QE durations will be required when having to make a longer putt (e.g. Vickers 2012), when putting to a smaller target, or with a smaller putter face.

The second aim was to adopt a more sensitive analysis relating the different proportions of the QE (early and late; Vine et al. 2013) to specific manipulations. Previous research has demonstrated that reductions in the late QE duration result in participants missing critical information regarding putter location and the putter-ball contact, leading to inferior performance (Vine et al. 2013). As such, the late QE is suggested to be responsible for the online control of movements (Vine et al. 2015). While exploratory, we suggested that a manipulation related to increasing the difficulty of making an optimal putter-ball impact (a putter insert) will likely influence the late proportion of the QE (online guidance of impact quality) to a greater extent than the early proportion of the QE.

Historically, research has focused on the QE’s relation to the pre-programming of movement parameters (Mann et al. 2011; Williams et al. 2002; Vickers 1996). Vickers (1996) postulated that movement parameters, including force and velocity, were programmed in the final fixation during the preparatory phase of movement. We suggested the manipulation of force production (length of putt) may influence the early portion of the QE (pre-programming swing length parameters). However, as stated above such investigation and hypotheses are largely exploratory due to the novelty of this work and limited examination of the QE proportions and specific movement parameters.

## Methods

### Participants

Thirty-four golfers (age: *M* = 21.35, *SD* = 4.04) with an average self-reported handicap of 7.2 (*SD* = 6.44) volunteered to take part in the experiment. All participants provided written informed consent, and local ethics committee approval was granted prior to testing.

### Manipulation of task difficulty

We manipulated the target size [large, 10 cm (3.9 in.) vs. small, 5 cm (1.9 in.)], length of the golf putt [short, 4 ft (1.2 m) vs. long, 8 ft (2.4 m)], and the size of the effective putter face using magnetic inserts [contact point: large, 1.7 cm (0.7 in.), 24 g vs. small, 0.6 cm (0.2 in.), 14 g].^1^ Varying these manipulations in a systematic fashion lead to the creation of eight conditions of increasing difficultly. The order of these eight conditions was randomised, and a Latin squares design was used to avoid order effects.^2^

### Apparatus

Participants putted using a standard length 90 cm steel-shafted blade style putter and standard size (4.27 cm diameter) white golf balls. In order to measure gaze behaviour, a lightweight Applied Science Laboratories (ASL; Bedford, MA) Mobile Eye Tracker XG was used to capture eye movements at 30 Hz (spatial accuracy of ± 0.5° visual angle; 0.1° precision). The Mobile Eye tracks the translation and rotation of the participant’s eye movements by means of the corneal reflection technique that gets superimposed as a fixation on the video footage of a scene camera. The gaze location is represented by a circular cursor that was set to reflect 1° of visual angle.

### Procedure

Participants read an information sheet, completed a demographic questionnaire, were fitted with an eye tracker and were allowed five familiarisation putts from 8 ft. Putts were taken on an artificial green and aimed towards a circular target projected onto the surface of the green using a Hitachi LCD mobile projector and Microsoft Powerpoint software. A projected target (rather than a hole) requires more precision in pace judgement than a normal sunken hole and was used to further increase task difficulty. The participants were provided with details relevant to each condition and were instructed to try to stop the ball on the projected target. In order to reduce a learning effect and to maintain the novelty of the task for each putt, the target was moved to one of three positions (left, centre or right). We also restricted feedback by removing the projected target just after putter-ball contact. Participants were then asked to face away from the target while the target reappeared and putts were measured. A total of 10 putts were executed in each condition, and rest periods were provided between conditions. The first five putts were then selected for gaze analysis in order to limit the potential for participants from making adjustments to overcome the manipulation of the task difficulty (e.g. Moore et al. 2012).

### Measures

#### Performance

The radial error (i.e. the two-dimensional Euclidean distance between the top of the ball and the edge of the target; in cm) was recorded using a tape measure after each putt (Vine et al. 2015). Error scores were then averaged (mean radial error) for each condition as a measure of performance.

#### Quiet Eye (QE)

The QE was calculated using Quiet Eye Solutions vision-in-action software (www.QuietEyeSolutions.com), which enabled momentary gaze location to be assessed in relation to the putter movement (also recorded by the mobile eye’s scene camera).

The QE was operationally defined for golf putting as the final fixation on the ball, with an onset prior to initiation of movement (backswing) and an offset when gaze deviates from the ball by more than 1° visual angle and for more than 100 ms (3 frames, i.e. 99.9 ms; Vine et al. 2015). The early phase of the QE (QE-early) started at QE onset and ended with the initiation of the backswing. The late phase of the QE (QE-late) started at the initiation of the backswing and finished when the putter contacted the ball or at QE offset (if prior to ball contact; Vine et al. 2015).

Duration measures were averaged for each participant’s first five trials. Due to technical errors in the data collection, four participants had to be removed and were not considered in data analyses.^3^ In the case where participants demonstrated no QE fixation, a zero value was entered for that trial (Williams et al. 2002).^4^ No QE fixation occurred due to the fixation onset starting after the backswing onset. However, if no QE fixation occurred due to technical difficulties the trial was excluded from further analysis.

### Data and statistical analysis

QE and performance data were subjected to 2 (target size) × 2 (length) × 2 (putter face) factorial analyses of variance (ANOVAs), with the alpha level set to < .05 and Greenhouse–Geisser corrections applied if sphericity assumptions were violated. Spearman’s rank-order correlations were also performed on QE duration and performance error measures in each of the eight conditions. Three univariate outliers classified as values more than 3.3 standard deviation units from the grand mean (Tabachnick and Fidell 1996) were Winsorized by changing the extreme raw score to a value 1% larger or smaller than the next most extreme score (as in Shimizu et al. 2011). Effect size was calculated using partial eta squared ($\eta_{\text{p}}^{2}$) for omnibus comparisons. All data analyses were conducted using IBM SPSS 20.0.

## Results

### Performance

**Fig. 1:**
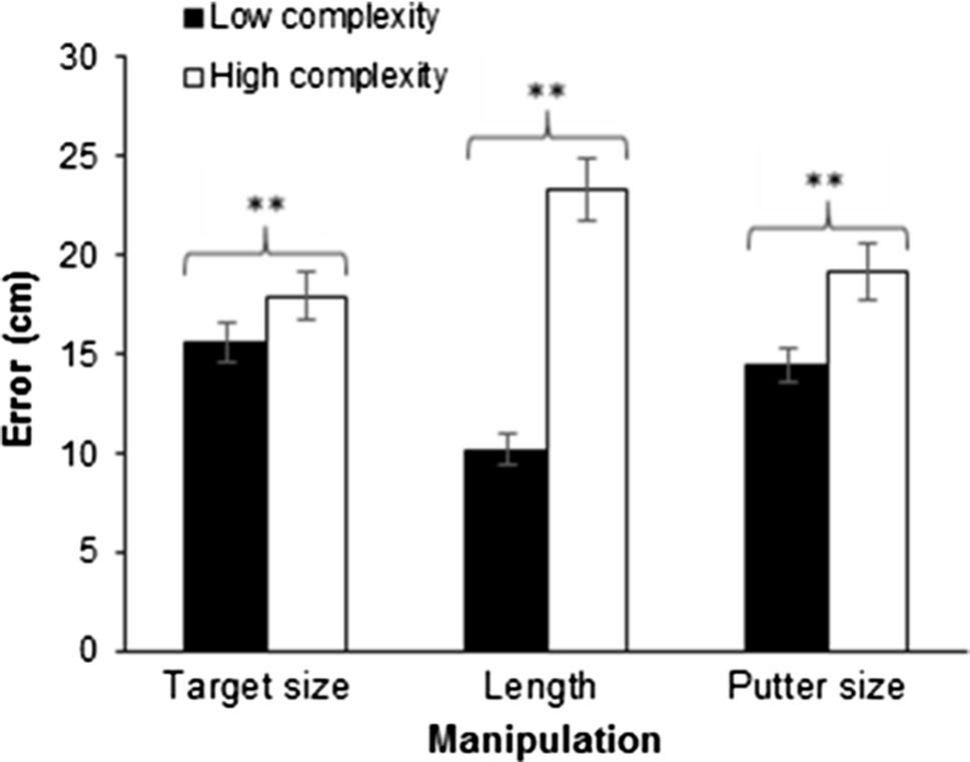
Performance error of the target size, length and putter face manipulations (mean ± SEM). Significant differences are denoted, **p < .01

ANOVA revealed significant main effects for target size [*F*(1,29) = 7.78, *p* = .009, $\eta_{\text{p}}^{2}$ = .21],^5^ length [*F*(1,29) = 90.11, *p* = .001, $\eta_{\text{p}}^{2}$ = .76] and putter face size [*F*(1,29) = 15.94, *p* = .001, $\eta_{\text{p}}^{2}$ = .39]. Participants’ radial error was larger for the more difficult iteration of each manipulation (see Fig. 1). No significant interactions were found (all *p*’s > .062, $\eta_{\text{p}}^{2}$ < .12).

### Quiet Eye

**Fig. 2:**
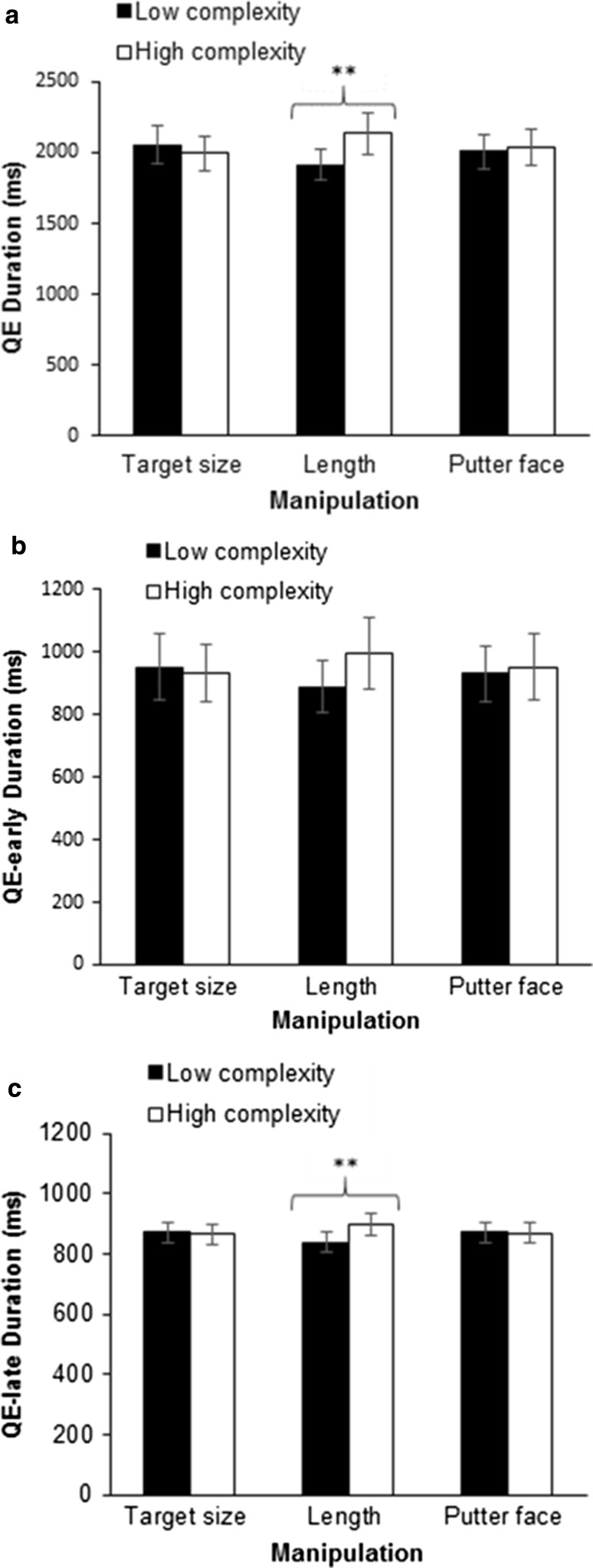
Total QE (**a**), QE-early (**b**) and QE-late (**c**) durations for each manipulation of target size, putt length and putter face size (mean ± SEM). Significant differences are denoted, **p < .01

For overall QE duration, ANOVA revealed non-significant main effects for target size [*F*(1,29) = 1.72, *p* = .200, $\eta_{\text{p}}^{2}$ = .06] and putter face size [*F*(1,29) = 0.53, *p* = .473, $\eta_{\text{p}}^{2}$ = .02]. However, a significant main effect for length [*F*(1,29) = 13.68, *p* = .001, $\eta_{\text{p}}^{2}$ = .32] was found (see Fig. 2a). A significant interaction was found for length and putter face [*F*(1,29) = 6.40, *p* = .017, $\eta_{\text{p}}^{2}$ = .18]. Follow-up *t* tests revealed that in the conditions where the putter face was small the longer putt had a significantly longer QE duration [*t*(29) = 4.20; *p* = .001]. No other significant interactions were found (all *p*’s > .169, $\eta_{\text{p}}^{2}$ < .18].

For QE-early, ANOVA revealed non-significant main effects for target size [*F*(1,29) = 0.19, *p* = .668, $\eta_{\text{p}}^{2}$ = .01], putter face size [*F*(1,29) = 0.32, *p* = .579, $\eta_{\text{p}}^{2}$ = .01] and for the length manipulation [*F*(1,29) = 4.06, *p* = .053, $\eta_{\text{p}}^{2}$ = .12] (see Fig. 2b). However, an interaction effect was found between length and putter face [*F*(1,29) = 7.12, *p* = .012, $\eta_{\text{p}}^{2}$ = .20]. Follow-up *t* tests revealed that in the conditions where the putter face was small the longer putt had a significantly longer QE duration [*t*(29) = 3.50; *p* = .002]. In long putting distance conditions, a small putter face had longer QE-early durations [*t*(29) = 2.18; *p* = .037]. No other significant interactions were found (all *p*’s > .096, $\eta_{\text{p}}^{2}$ < .09).

For QE-late, ANOVA revealed non-significant main effects for target size [*F*(1,29) = 0.21, *p* = .654, $\eta_{\text{p}}^{2}$ = .01] and putter face [*F*(1,29) = 0.03, *p* = .862, $\eta_{\text{p}}^{2}$ = .01]. There was a significant main effect for length [*F*(1,29) = 13.02, *p* = .001, $\eta_{\text{p}}^{2}$ = .31] (see Fig. 2c). No significant interactions were found (all *p*’s > .223, $\eta_{\text{p}}^{2}$ < .05).

### Quiet Eye: performance relationship

In four conditions, there were weak negative correlations (Con3. large target, long length, large putter; Con5. small target, short length, large putter; Con6. small target, short length, small putter; Con7. small target, long length, large putter), which were not statistically significant [all *r*_s_’s > − .01, all *p*’s > .308]. In the remaining conditions, there were weak positive correlations, three of which were not statistically significant (Con1. large target, short length, large putter; Con2. large target, short length, small putter; Con8; small target, long length, small putter) [all *r*_s_’s > .15, all *p*’s > .184] and one was statistically significant (Con4. large target, long length, small putter) [*r*_s_ = .39, *p* = .032].

## Discussion

The aim of this study was to examine the response programming explanation of the QE by manipulating the difficulty of a golf-putting task. Task difficulty was successfully altered in all three manipulations (force, impact and target line), as performance error was higher with more difficult iterations of each manipulation. The lack of any significant interaction effects would suggest a floor effect for performance. Furthermore, while it would seem that the size of the putter insert contributed to the change in performance, its additional weight may also have had an effect on performance. At present, it is not possible to determine the true reason for changes in performance.

The results for the QE measures were more complex, reflecting the fact that performance and QE measures might not necessarily have a monotonic relationship. The manipulation of target size had no impact on QE, perhaps because the aiming point (the centre of the target circle) was the same in both conditions. We did find that the QE was sensitive to changes in requirements for accurate force production; as the length of putts increased so did overall QE and QE-late durations. This strategy does provide more time for online control of movements (e.g. Lam et al. 2010); however, it may be a side effect of the longer putting stroke used to propel the ball to the further target (Williams et al. 2002).^6^ Yet increased force demands does not necessarily require a longer swing, swing durations can be maintained while increasing force and amplitude (Delay et al. 1997). Extended swing durations could reflect an intentional strategy to provide more time for online control of movements (Fitts 1954; Corben et al. 2011; Lam et al. 2010).

Nonetheless, the most notable finding is the length by putter face interaction for overall QE and QE-early durations, which, while not fully supporting our initial hypotheses, do support Vickers’ (1996) proposition that movement parameters are programmed prior to movement initiation. Taken together, the findings suggest that participants took longer QE-early durations to prepare for the most difficult tasks (long putt and small putter face). It is unclear from the results whether QE and QE-early increased due to the need for an objective rescaling of movement parameters or due to a subjective need to pause and prepare psychologically for the task, both provoking the allocation of additional cognitive resources.

Recent research has focused on the importance of QE-late durations for controlling movements online (Vine et al. 2013; 2015); however, the current findings refute this idea and provide support for the role of QE in pre-programming movements (as proposed by Vickers 1996). However, it is possible that the mechanisms causing changes in QE in this study (in response to task difficulty) are different to the mechanisms that cause changes to QE in the previous research from Vine and colleagues, which focused on manipulating state anxiety. Such differences perhaps provide the opportunity for future research to explore more theoretical explanations for the role of QE in supporting performance. For example, Vine et al. (2013, 2015) suggested that anxiety made it more difficult to maintain goal-directed focus (based on the prediction of Eysenck et al.’s (2007) Attentional Control Theory) and that this control was most likely to break down late in the swing, as target related disruptions became most salient. Also, contrary to previous research (e.g. Vine et al. 2013, 2015), the longer QE durations found in the more difficult conditions were not associated with superior performance. One condition revealed that longer QE durations were related to less accurate performance, suggesting that increased QE in response to task difficulty was not functional for these experienced golfers. More specifically, the additional task parametrisation before movement did not translate into better movements.

Nevertheless, the increased cortical investment allocated to movement preparation that accompany longer early QE durations (Mann et al. 2011; Moran et al. 2016) may have prevented even greater performance decrements. As such, the longer QE durations may in fact have had a positive, insulating effect, although this is difficult to determine using the current research design. It is impossible to unpick the cumulative and opposing influences that extended QE durations and increased task demands have on performance. Future research is needed to better understand precisely why increased difficulty causes a change in QE duration and to decipher how the QE can be associated with more difficult (and hence less accurate) performance on the one hand and superior performance on the other.

To conclude the current study builds on previous research by indicating that QE is sensitive to the programming of specific task parameters, supporting the response programming function of the QE. Specifically, the importance of the early QE rather than late QE proportions indicates that different mechanisms may be at play when putting under different circumstances, such as anxiety. Nonetheless, the increases in QE do not seem functional in terms of supporting improved performance but may provide an insulating effect. Further research is needed to explore QE’s relationship with performance under conditions of increased difficulty.

## Footnotes

1 While varying the size of the contact point was the intended manipulation of difficulty, the insert also changed the weight of the putter.

2 No significant main effects were found for the condition order [*F*(7,203) = 0.76, *p* = .551, $\eta_{\text{p}}^{2}$ = .03].

3 Due to technical errors, all tracking data were lost for two participants and tracking data in one condition were lost for two additional participants. As such, these participants were not entered into the ANOVA for all variables and this is reflected in the degrees of freedom in the results section. In addition, across conditions 1, 2 and 3 there were 7 participants that had average QE durations taken from less than 5 putts (no less than 3; again due to technical errors).

4 Out of the possible 620 trials, no fixations occurred for 10 trials (1.6%). Levels of significance were unaffected when removing zeros from QE and QE-late analysis. QE-early analysis brought the significance of the length manipulation to < .05 (*p* = .047).

5 It is possible that the different target sizes contributed to the differences in radial error. When the target sizes were accounted for (i.e. Putts that stopped within the circumference of the large target were excluded) additional analysis reveals participants were still significantly less accurate in the small target condition (large target *M* = 17.36 cm, SD = 1.06; small target *M* = 21.00 cm, SD = 1.21) [*F*(1,29) = 19.56, *p* = .001, $\eta_{\text{p}}^{2}$ = .40]. We thank an anonymous reviewer for this suggestion.

6 QE duration relative to swing duration revealed non-significant main effects for target size [*F*(1,29) = 0.24, *p* = .627], putter face size [*F*(1,29) = 0.01, *p* = .957] and for the length manipulation [*F*(1,29) = 0.01, *p* = .930]. No significant interactions were found (*p*’s *>* .303).
